# Long‐Term Health‐Related Quality of Life Post‐COVID‐19 ICU: A Synthesis of Key Findings

**DOI:** 10.1002/hsr2.70838

**Published:** 2025-05-19

**Authors:** John Patrick C. Toledo

**Affiliations:** ^1^ Department of Theology and Religious Education De La Salle University Manila Philippines

Dear Editor,

I've read with great interest the study by Samuelsson, Hussain, Drummond, and Persson, which scientifically explores the long‐term health‐related quality of life (HRQoL) and the prevalence of anxiety and depression symptoms in individuals 1 year after intensive care unit (ICU) admission for COVID‐19. The results indicate the importance of continuous follow‐up care and assistance for these patients by highlighting serious mental health issues and reduced HRQoL. The findings of the study are especially pertinent for determining the variables linked to lower HRQoL and mental health problems, which will direct focused measures to enhance the well‐being of COVID‐19 intensive care unit survivors.

The study assessed HRQoL using the EuroQol 5 Dimensions 3 Levels (EQ‐5D‐3L) questionnaire and the EuroQol visual analogue scale (EQ‐VAS). A year after ICU discharge, a substantial proportion of participants reported moderate to extreme problems with pain/discomfort (69%) and anxiety/depression (51%). The mean EQ‐5D‐3L value index was 0.83, and the median EQ‐VAS score was 70^1^. Diabetes mellitus was linked to poor self‐care, while longer ICU stays were linked to pain/discomfort and a lower HRQoL in daily activities. Furthermore, lower HRQoL in terms of pain and discomfort was linked to younger age and female sex.

This study contributes to the long‐term functional and psychosocial outcomes of COVID‐19 intensive care unit survivors. It highlights the need for specialized support by identifying particular risk factors for depression, such as female sex. A major public health concern, according to the report, is the high prevalence of anxiety (38.1%) and depression (35.2%) 1 year after ICU admission^1^. These results highlight the significance of ongoing mental health care and long‐term monitoring for patients hospitalized to intensive care units.

The study highlights the necessity of ongoing rehabilitation and mental and physical health care during the first year following ICU discharge, particularly for individuals with diabetes. The study indicates that specific therapies and support networks are essential due to the substantial influence on HRQoL, especially in domains such as pain/discomfort and anxiety/depression. Healthcare professionals may provide more individualized and efficient care by acknowledging the unique difficulties faced by female patients and those who require longer stays in the intensive care unit. This will ultimately improve the long‐term quality of life for COVID‐19 ICU survivors.

## Author Contributions

**John Patrick C. Toledo:** conceptualization, methodology, and writing – original draft.

## Ethics Statement

Ethical standards are followed in the research.

## Conflicts of Interest

The author declares no conflicts of interest.

## Data Availability

The data that supports the findings of this study is available from the Health Sciences Reports and other relevant materials.

## Endnotes

1 Samuelsson, C. M., Hussain, N., Drummond, A., and Persson, C. U. (2025). Health‐Related Quality of Life 1 Year After Intensive Care Unit Admission for COVID‐19: A Retrospective, Cross‐Sectional, Longitudinal Observational Study. *Health Science Reports*, 8, no. 2: e70507. https://doi.org/10.1002/hsr2.70507

